# The Effect of Antibiotic and Nonantibiotic Drugs on Plasmid‐Mediated Bacterial Conjugation

**DOI:** 10.1155/ijm/3323758

**Published:** 2026-05-06

**Authors:** Marcus Daitey Larnyoh, Seth Kwabena Amponsah, Abigail Offei, Emmanuel Kwaku Ofori, Ofosua Adi-Dako, Awo Afi Kwapong

**Affiliations:** ^1^ Department of Medical Pharmacology, University of Ghana Medical School, Accra, Ghana, ug.edu.gh; ^2^ Department of Anaesthesia and Intensive Care, 37 Military Hospital, Accra, Ghana; ^3^ Department of Chemical Pathology, University of Ghana Medical School, Accra, Ghana, ug.edu.gh; ^4^ Department of Pharmaceutics and Microbiology, University of Ghana School of Pharmacy, Legon, Ghana, ug.edu.gh

**Keywords:** antibiotic, conjugation, horizontal gene transfer, plasmid, resistance

## Abstract

**Background:**

The clinical utility of antibiotics has been eroded by the emergence of antibiotic resistance. One major mechanism by which microorganisms develop resistance to antibiotics and nonantibiotics is by horizontal gene transfer (HGT) via plasmid‐mediated conjugation.

**Aim:**

To investigate the impact of specific antibiotics and nonantibiotics on plasmid‐mediated bacterial conjugation and elimination.

**Methods:**

The minimum inhibitory concentration (MIC) of the selected antibiotics and nonantibiotics was determined for *Escherichia coli* (ATCC 25922) using the broth microdilution method. The anticonjugant activities of the test drugs were assessed using the liquid conjugation assay on plasmids IncN plasmid pKM101, IncP plasmid pUB307, and IncW plasmid R7K in *E. coli*. Additionally, the ability of the test drugs to eliminate and/or cure plasmids was determined

**Results:**

At subinhibitory concentrations, several antibiotics—including azithromycin, doxycycline, and ceftriaxone—and nonantibiotic pharmaceuticals, such as amlodipine and propranolol, facilitated the horizontal transfer of plasmid‐borne antibiotic‐resistant genes in a plasmid‐specific manner. Amlodipine notably enhanced the conjugative transfer of IncN plasmid pKM101 by 2.52‐fold and the IncP plasmid pUB307 by 4.23‐fold. Propranolol also increased the transfer of IncN plasmid pKM101, albeit modestly (1.14‐fold). Plasmid curing activity was broad and nonselective in the case of amlodipine, doxycycline, glibenclamide, and levofloxacin, whereas propranolol exhibited plasmid‐specificity curing activity, particularly against IncW plasmid R7K.

**Conclusion:**

These findings demonstrate that antibiotics and nonantibiotic drugs can exert dual, context‐dependent effects, simultaneously promoting plasmid transfer while eliminating specific plasmids. This plasmid‐specific interplay highlights the complexity of drug–microbe interactions and underscores the need for careful evaluation of their roles in antimicrobial resistance dynamics.

## 1. Introduction

Antimicrobial resistance is increasing, necessitating the implementation of measures to address this worldwide public health issue [1]. This appears to be more prevalent in low‐ and middle‐income countries (LMICs) where there is an already existing disparity in availability of appropriate medical equipment, diagnostic tools, and high‐quality antimicrobials [2–4].

Horizontal gene transfer (HGT) refers to the process through which microorganisms of the same or different species acquire new genetic material from outside their clonal lineage [5]. HGT is especially important during infections and outbreaks as it aids in transferring genes that may encode for virulence and biofilm‐forming capability [6]. The actual contribution of antibiotics and nonantibiotics to HGT has been a subject of debate [7, 8]. This debate is compounded by the many methods available for carrying out in vitro plasmid conjugation assays, which can involve varying experimental conditions [9–11].

In [12]. showed that, *Escherichia coli* exposed to cefotaxime (CTX), resulted in considerable increase in the conjugative transfer of the *bla*_CTX−M−1_ encoding IncI1 plasmid (IncI1/pST49/CTX‐M‐1) in an SOS‐independent manner [12]. In a similar study that expanded the number of *E. coli* strains and number of antibiotics, plasmid transfer was increased by antibiotic exposure in 8 (30.8%) out of the 26 strains, all of which carried plasmids belonging to the incompatibility groups IncI1 and IncFII [13].

A study indicated that over 200 nonantibiotic medications may exert antibiotic‐like actions on specific human gut bacterial strains [14]. Common among these drugs are antidepressants, analgesics, anticancer drugs, lipid‐lowering drugs, and antihypertensives. These drugs can enhance the transfer of antimicrobial resistant genes carried on self‐transmissible plasmids [15]. Wang et al. *[* *16* *]* showed that, at a concentration as low as 0.005 mg/L, naproxen caused the conjugative transfer of the plasmid, RP4 from *E. coli* to *P. putida* to increase by a factor of four [16, 17].

It is imperative to investigate further the phenomenon of HGT using antibiotics and nonantibiotic drugs that are often used in LMICs, as there appears to be a paucity of data. We, therefore, investigated the effect of frequently used antibiotics and nonantibiotics on bacterial plasmid‐mediated conjugation and elimination/curing using in vitro assays. The study protocol used plasmids from incompatibility groups: IncN (pKM101), IncP (pUB307), and IncW (R7K).

## 2. Materials and Methods

### 2.1. Materials

Mueller–Hinton broth (MHB) and Luria–Bertani (LB) growth media were puchased from Sigma (United States). Normal saline (0.9% w/v), standard test samples—including antibiotics (levofloxacin, ceftriaxone, cefuroxime, meropenem, doxycycline, clindamycin, ceftazidime, metronidazole, ampicillin, and ciprofloxacin), antidiabetics (glimepiride, glibenclamide, and metformin), and antihypertensives (lisinopril, methyldopa, losartan, and amlodipine)—were obtained from Thermo Fisher Scientific (United States). Additional materials included 96‐well microtiter plate, multiple channel pipette, universals and bijous, and 3‐(4,5‐Dimethylthiazol‐2‐yl)‐2,5‐diphenyltetrazolium bromide (MTT). All other chemicals and reagents were sourced from reputable suppliers.

The *E. coli* cells containing plasmids and their incompatibility groups (Inc) utilized in this work are presented in Table 1. All these were obtained from cryoprecipitates supplied by Dr. Awo Afi Kwapong, School of Pharmacy, University of Ghana.

**Table 1:** Summary of *E. coli* strains and plasmids used and their incompatibility group.

Strain	Plasmid	Incompatibility (Inc) group	Resistance markers
Donor			
WP2	pKM101	N	Ap
K12 JD173	pUB307	P	Km, Tc
K12 J53‐2	R7K	W	Ap, Sm, Sp
Recipient			
ER1793	—	—	Sm
JM109	—	—	Nal

Abbreviations: Ap, ampicillin; Km, kanamycin; Nal, nalidixic acid; Sm, streptomycin; Sp, spectinomycin; Tc, tetracycline.

### 2.2. Assessment of the Minimum Inhibitory Concentration (MIC)

The broth microdilution assay was employed to ascertain the MIC as an indicator of the antibacterial efficacy of the selected drugs. This method was previously described by [18]. MICs were determined using *E. coli* ATCC 25922, a standard quality‐control reference strain, to ensure reproducibility and to establish intrinsic antimicrobial activity independent of plasmid carriage, thereby enabling consistent selection of subinhibitory concentrations for conjugation assays. Briefly, *E. coli* (ATCC 25922) was cultivated on nutrient agar slants and incubated for 18 h at 37°C. An inoculum suspension was prepared in normal saline (0.9% w/v) and calibrated to the 0.5 McFarland turbidity standard. MHB and the selected antibiotics (levofloxacin, ceftriaxone, cefuroxime, meropenem, doxycycline, clindamycin, ceftazidime, metronidazole, etc), antidiabetics (glimepiride, glibenclamide, and metformin) and antihypertensives (lisinopril, methyldopa, losartan, and amlodipine) (with varying initial concentrations ranging from 512 to 8 *μ*g/mL as shown in Tables 2, 3, and 4) were then serially diluted across a 96‐well microtiter plate, after which the bacteria inoculum suspension was dispensed into each well to achieve a final inoculum concentration of 0.5 × 10^5^ CFU/mL. This was incubated for 18 h at 37°C, after which the MICs were determined. Visual inspection was conducted following the addition of 1 mg/mL methanolic solution of 3‐[4,5‐dimethylthiazol‐2‐yl]‐2,5‐diphenyl tetrazolium bromide (MTT) into each well of the test plate (Figure S1), which was subsequently incubated at 37°C for 20 min [19]. Columns 11 and 12 of the assay plates served as growth and sterility controls, respectively. Rows A1–F1 of each plate contained the test samples, whereas Rows G1 and H1 contained the control drug (ciprofloxacin). The experiment was conducted twice in three separate independent trials.

**Table 2:** Start concentrations of the selected antibiotics and comparator against *Escherichia coli* ATCC 25922.

Antibiotic	Initial concentration (mg/L)
Doxycycline	8
Clindamycin	16
Azithromycin	8
Trimethoprim	8
Sulfamethoxazole	8
Furazolidone	8
Tinidazole	8
Metronidazole	16
Cefuroxime	16
Cefpodoxime	8
Ceftriaxone	8
Clarithromycin	8
Cefixime	16
Meropenem	16
Levofloxacin	8
Flucloxacillin	8
Ciprofloxacin (control)	8

*Note:* Two‐fold (serial) dilution scheme used.

**Table 3:** Start concentrations of the selected antidiabetics and comparator against *Escherichia coli* ATCC 25922.

Antidiabetics	Initial concentration (mg/L)
Metformin	512
Gliclazide	512
Glibenclamide	512
Glimepiride	512
Ciprofloxacin (control)	8

*Note:* Two‐fold (serial) dilution scheme used.

**Table 4:** Start concentrations of the selected antihypertensives and comparator against *Escherichia coli* ATCC 25922.

Antihypertensive	Initial concentration (mg/L)
Amlodipine	512
Losartan	512
Propranolol	512
Lisinopril	512
Furosemide	512
Bendroflumethiazide	512
Methyl dopa	512
Ciprofloxacin (control)	8

*Note:* Two‐fold (serial) dilution scheme used.

### 2.3. Determination of Plasmid Conjugation Inhibition Properties of Test Samples

Plasmid carriage can influence bacterial fitness and limit their adaptability in new hosts [20, 21]. To assess the effect of selected compounds on plasmid transfer, *E. coli* donor strains (WP2, K12 JD173, and K12 J53‐2) harboring plasmids pKM101 (IncN), pUB307 (IncP), and R7K (IncW), respectively, were paired with compatible recipient strains ER1793 and JM109 (Table 1). These strains were chosen for their well‐established conjugation efficiency and plasmid maintenance characteristics [22, 23]. The conjugation assay followed a liquid‐mating protocol adapted from Rice and Bonomo [24], with minor modifications as outlined by Kwapong [25] and Gibbons [26]. Briefly, two to three isolated colonies of both donor and recipient strains were separately inoculated into 5 mL of LB broth and incubated at 37°C overnight. The resulting cultures were serially diluted in sterile normal saline using 96‐well microplates. From the diluted cultures, 20‐*μ*L volumes from dilutions 10^−4^, 10^−5^, 10^−6^, and 10^−7^ were plated on selective MacConkey agar and incubated at 37°C for 18 h. Colony counts were then used to estimate the viable bacterial load using the following formula:

Colony−forming units CFU=average colony countvolume plated×dilution factor

Following colony‐forming unit (CFU) determination (Table S1), donor and recipient cultures were mixed in equal volumes (20 *μ*L each) in 160 *μ*L of LB broth containing either the test compound or control (LB only) in a 96‐well plate. The plate was incubated for 16–20 h at 37°C. After incubation, transconjugants and donor cells were enumerated by plating aliquots onto MacConkey agar containing appropriate antibiotics. The test compounds were evaluated at a concentration equal to one‐quarter of their MIC values against *E. coli* ATCC 25922. Selective markers used for differentiation included amoxicillin (50 mg/L), streptomycin (20 mg/L), kanamycin sulphate (30 mg/L), and nalidixic acid (30 mg/L). A negative control containing donor, recipient, and medium only (no test compound) was used for baseline comparison. Conjugation inhibition of test compounds was inferred by comparing the transconjugant CFU count with donor counts and expressed as a percentage relative to the control transconjugant and control donor as shown in the formula below. Each assay was conducted in duplicate and repeated in three independent experimental runs. Results were reported as mean ± standard deviation (SD). The experimental model assumes random conjugation events between freely suspended donor and recipient cells.

Conjugation inhibition %=Conjugation frequency of test compoundConjugation frequency of control×100

### 2.4. Plasmid Elimination Assay

The ability of test samples to eliminate plasmids from bacterial hosts was assessed using a plasmid curing method previously outlined by Hooper et al. [27], with slight adjustments based on the modifications described by Kwapong [25] and Gibbons [26]. To verify the presence of plasmids prior to the experiment, donor strains were streaked on MacConkey agar supplemented with the relevant antibiotics. After incubating at 37°C for 18 h, two to three well‐isolated colonies were selected and inoculated into 5 mL of LB broth. The cultures were grown overnight under the same temperature conditions.

Before the actual assay, bacterial density was estimated by determining the number of CFUs. Once cell concentrations were established, 20 *μ*L of the overnight donor culture was transferred into wells of a 96‐well microplate containing 180 *μ*L of LB broth along with the test compound. The microplates were incubated for another 18 h at 37°C. After this incubation period, the cultures were serially diluted in sterile saline, and 20 *μ*L of each dilution was plated onto two types of media: plain MacConkey agar (for total viable bacterial count) and MacConkey agar containing antibiotics (to assess plasmid retention/bearing cells). Plates were again incubated at 37°C for 18 h. Colony counts were recorded for both types of agar. The difference between the total number of colonies on nonselective agar and those on antibiotic‐containing agar was used to estimate plasmid loss. The same subinhibitory concentration used in the conjugation inhibition assay (one‐fourth MIC) was applied in this assay to ensure consistency. Each test sample was run alongside a positive control (promethazine) and a negative control (donor strain without any test agent), which reflected the natural rate of plasmid retention. All experimental conditions were performed in biological triplicates. The extent of plasmid elimination was quantified using the formula:

Plasmid elimination %=1− CFU of plasmid−bearing cells Total CFU×100

Presenting the results as a percentage allowed for a uniform comparison of plasmid elimination efficiency across different samples and conditions. This standardization also enabled clearer interpretation of how each compound influenced the stability and persistence of plasmids within host bacteria.

### 2.5. Statistical Analysis

All experiments were conducted in triplicates and the data were expressed as mean with SD of mean. Data were summarized into tables and bar charts using Microsoft Excel and GraphPad Prism 10 (GraphPad Software, LLC). Statistical analysis was conducted using the one‐way ANOVA and post hoc Tukey′s test to assess differences between the control conjugation transfer frequency and the test samples. Results with *p* value of < 0.05 were deemed statistically significant.

## 3. Results

### 3.1. Effect of the Selected Antibiotics, Antidiabetics, and Antihypertensives on Bacterial Growth

To assess the growth inhibitory effects of the selected test samples (antibiotics, antidiabetics, and antihypertensives) on bacteria and determine an appropriate concentration for the anticonjugation assay, the antibiotics and nonantibiotics were tested against susceptible Gram‐negative standard isolate *E. coli* ATCC 25922. Tables 5, 6, and 7 show the MICs for the tested antibiotics and nonantibiotics. The inhibitory activity of the test antibiotics ranged from > 16 to 0.125 mg/L, whereas the nonantibiotics (antidiabetics and antihypertensives) exhibited MIC values from > 512 to 256 mg/L against the evaluated bacteria. Overall, the nonantibiotic pharmaceuticals showed no notable in vitro inhibitory activity against *E. coli* ATCC 25922, whereas the screened antibiotics demonstrated potent inhibition. Among them, doxycycline exhibited the highest inhibitory activity (0.125 mg/L) against *E. coli* ATCC 25922, though it was less potent than the control antibiotic, ciprofloxacin, which had an MIC of 0.031 mg/L.

**Table 5:** Minimum inhibition concentrations (MICs) of the selected antibiotics and comparator against *Escherichia coli* ATCC 25922^a^.

Antibiotic	MIC (mg/L)
Doxycycline	0.125
Clindamycin	> 16
Azithromycin	4
Trimethoprim	> 8
Sulfamethoxazole	> 8
Furazolidone	> 8
Tinidazole	> 8
Metronidazole	> 16
Cefuroxime	8
Cefpodoxime	> 8
Ceftriaxone	0.5
Clarithromycin	4
Cefixime	>16
Meropenem	>16
Levofloxacin	4
Flucloxacillin	> 8
Ciprofloxacin (control)	0.031

^a^Susceptible standard strain. The results shown are the average of three independent biological replicates.

**Table 6:** Minimum inhibition concentrations (MICs) of the selected antidiabetics and comparator against *Escherichia coli* ATCC 25922^a^.

Antidiabetics	MIC (mg/L)
Metformin	> 512
Gliclazide	> 512
Glibenclamide	256
Glimepiride	> 512
Ciprofloxacin (control)	0.031

^a^Susceptible standard strain. The results shown are the average of three independent biological replicates.

**Table 7:** Minimum inhibition concentrations (MICs) of the selected antihypertensives and comparator against *Escherichia coli* ATCC 25922^a^.

Antihypertensive	MIC (mg/L)
Amlodipine	256
Losartan	> 512
Propranolol	512
Lisinopril	> 512
Furosemide	> 512
Bendroflumethiazide	> 512
Methyl dopa	> 512
Ciprofloxacin (control)	0.031

^a^Susceptible standard strain. The results shown are the average of three independent biological replicates.

### 3.2. Effect of the Selected Antibiotics, Antidiabetics, and Antihypertensives on Conjugal Transfer of Plasmids

To examine the anticonjugant activity of the selected antibiotics, antidiabetics, and antihypertensives, plasmids from different incompatibility groups (IncN plasmid pKM101, IncP plasmid pUB307, and IncW plasmid R7K) were used to evaluate their specificity in inhibiting conjugation in *E. coli*. Considering their MICs against *E. coli* ATCC 25922 (a susceptible reference strain) (Tables 5, 6, and 7), the selected antibiotics, antidiabetics, and antihypertensives were tested at a subinhibitory concentration of a quarter (0.25) of the MIC. The effect of the selected antibiotics, antidiabetics and antihypertensives on the conjugal transfer of the test plasmids is shown in Figure 2. The test samples exhibited inhibitory activities ranging from complete reduction in conjugation frequency (0%, considered active) and inhibition of conjugation frequency to < 10% (also considered active), to 10%–50% (moderately active) and > 50% (considered inactive or promoting conjugation) (Tables S2, S3, and S4).

### 3.3. Plasmid Elimination Effect of Selected Antibiotics, Antidiabetics, and Antihypertensives

**Figure 1:**
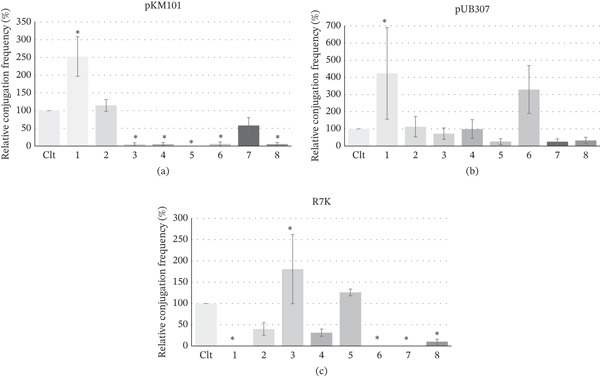
Effect of selected antibiotics and nonantibiotics on the conjugal transfer of (a) IncN plasmid pKM101, (b) IncP plasmid pUB307, and (c) IncW plasmid R7K, expressed as a percentage relative to the control without test compound (Clt). The antibiotics and nonantibiotics were tested at the following subinhibitory concentrations: amlodipine (**1**) (64 mg/L); propranolol (**2**) (128 mg/L); azithromycin (**3**) (1 mg/L); clarithromycin (**4**) (1 mg/L); doxycycline (**5**) (0.031 mg/L); ceftriaxone (**6**) (0.125 mg/L); glibenclamide (**7**) (64 mg/L); and levofloxacin (**8**) (1 mg/L). Values represent the mean ± standard deviation of at least three experiments measured by the liquid conjugation assay. ∗*p* < 0.05 (compared with the control).

To determine that the observed anticonjugant activity was not a result of conjugative plasmid elimination, donor cells were cultured in the presence of the test antibiotics and nonantibiotics; a plasmid elimination assay was conducted. Figure 1 shows the effect of the test antibiotics and nonantibiotics on conjugative plasmids. The test antibiotics and nonantibiotics showed varied plasmid‐curing activities, with elimination percentages ranging from 37.2*%* ± 13.4*%* to 0.4*%* ± 0.3*%* against the test plasmids. Amlodipine (64 mg/L), levofloxacin (1 mg/L), and promethazine (16 mg/L) were the only test samples that demonstrated moderate plasmid‐eliminating properties against all test plasmids, with plasmid‐eliminating percentages exceeding 10%. Doxycycline (0.03 mg/L) and glibenclamide (64 mg/L) showed moderate plasmid‐eliminating properties against plasmids pUB307 (IncP) and R7K (IncW) but not against pKM101 (IncN). The drugs with the lowest plasmid‐eliminating activity, showing elimination percentages below 5.5% or no plasmid curing effect against all test plasmids, were azithromycin, clarithromycin, and ceftriaxone (Tables S5, S6, and S7).

## 4. Discussion

Research data suggest that nonantibiotic agents may exert antibiotic‐like effects on certain human gut bacterial strains [7, 14]. Notably, antihypertensives, analgesics, anticancer drugs, lipid‐lowering agents, and antidepressants have been implicated. These drugs can facilitate the transfer of antimicrobial resistant genes via self‐transmissible plasmids [15]. Antibiotics, even at suboptimal concentrations, exert selective pressure on bacteria, potentially driving antimicrobial resistance. This phenomenon has been observed in clinical settings, within communities, and in the environmental context [28, 29]. In light of this, we investigated the effects of commonly used antibiotics, antidiabetics, and antihypertensives on bacterial plasmid‐mediated conjugation and elimination/curing in *E. coli* using in vitro assays.

The initial findings of this study indicated that the test samples exhibited varying degrees of bacterial growth inhibition. The selected antibiotics demonstrated antibacterial activity ranging from 0.125 to > 16 mg/L, whereas the tested antidiabetic and antihypertensive agents showed antibacterial activity ranging from 256 to > 512 mg/L against susceptible *E. coli* ATCC 25922 (Tables 5, 6, and 7). Among the test samples, doxycycline (MIC 0.125 mg/L, Figure S1) exhibited the highest antibacterial potency, though it was not as effective as the control drug ciprofloxacin (MIC 0.031 mg/L). As expected, the tested nonantibiotic agents were less potent than the antibiotics, likely due to their limited mechanisms of action, potential susceptibility to bacterial efflux systems, and nonspecific interactions with bacterial cells [30, 31].

**Figure 2:**
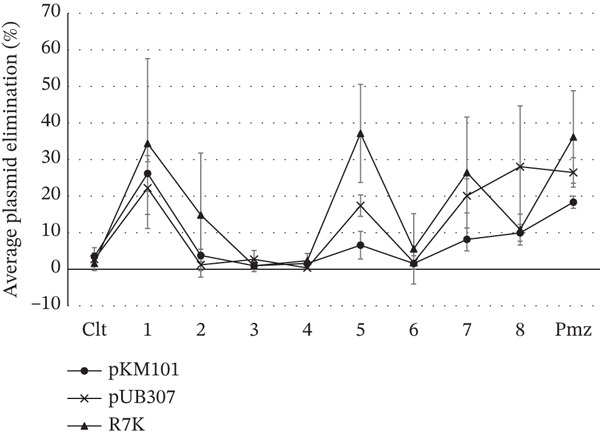
Plasmid elimination activity of the selected antibiotics and nonantibiotics. The antibiotics and nonantibiotics were tested at the following subinhibitory concentrations: amlodipine (**1**) (64 mg/L); propranolol (**2**) (128 mg/L); azithromycin (**3**) (1 mg/L); clarithromycin (**4**) (1 mg/L); doxycycline (**5**) (0.031 mg/L); ceftriaxone (**6**) (0.125 mg/L); glibenclamide (**7**) (64 mg/L); and levofloxacin (**8**) (1 mg/L). The negative control was **Clt** (without test sample), and the positive control was promethazine (**Pmz**; 16 mg/L). Values represent the mean ± standard deviation of at least three independent experiments measured by the plasmid elimination assay.

In the anticonjugal and antiplasmid activity studies, subinhibitory concentrations of the antibiotics and nonantibiotics were used. At these concentrations, bacterial growth is not inhibited; however, certain physicochemical changes occur within the bacteria, which can induce or enhance conjugative transfer and/or elimination of plasmids [32]. Compounds that reduced conjugation frequency without evidence of plasmid elimination suggest a possible inhibitory effect on the conjugation process, such as pilus formation, mating pair formation, DNA transfer machinery, *tra* genes, or conjugation proteins [32]. Conversely, compounds that showed plasmid elimination activity probably reduced conjugation indirectly by removing the conjugative plasmid from donor cells. The findings from this study revealed that amlodipine (**1**), at a concentration of 64 mg/L, selectively inhibited the conjugative transfer of plasmid R7K (IncW) while significantly increasing conjugation of plasmids pKM101 (IncN) and pUB307 (IncP) (Figure 1). In the presence of amlodipine (**1**), no conjugation of plasmid R7K was observed (Figure 1, Table S4), which may be due to its broad plasmid curing activity against all test plasmids (Figure 2). Specifically, amlodipine (**1**) eliminated plasmid R7K in *E. coli* K12 J53‐2 by 34.3*%* ± 23.2*%*, a higher elimination rate compared with the other test plasmids. This may account for the absence of conjugation observed for this plasmid. Amlodipine, a calcium channel blocker, has been reported to alter bacterial membrane properties and influence bacterial adhesion [33, 34]. These modifications can facilitate the formation of conjugate pili, thereby promoting plasmid conjugation and transfer. This may explain the observed increase in plasmid conjugation in pKM101 and pUB307 (conjugation frequencies > 120%) (Figure 1).

At a concentration of 128 mg/L, propranolol (**2**) enhanced the conjugative transfer of plasmids pKM101 and pUB307, with transfer frequencies exceeding 120% (Figure 1, Tables S2 and S3). This finding aligns with previous reports indicating that propranolol may promote plasmid conjugation by inducing oxidative stress, altering the bacterial cell envelope and/or modifying membrane fluidity; factors that collectively enhance the formation of conjugative pili [17]. Conversely, propranolol (**2**) moderately inhibited the conjugative transfer of plasmid R7K (IncW), reducing transfer frequency to 39.98*%* ± 14.70*%* (Figure 1). This moderate inhibition may be attributed to the partial elimination of plasmid R7K in *E. coli* K12 J53‐2, with a curing rate of 14.81*%* ± 16.97*%* (Figure 2).

At a concentration of 1 mg/L, azithromycin (**3**) showed varied effects on the conjugative transfer of the test plasmids, with no marked plasmid eliminating activity observed (Figure 2). It significantly inhibited the conjugal transfer of plasmid pKM101 (IncN), reducing the transfer frequency to 4.89*%* ± 4.38*%*. In contrast, it showed no notable inhibitory effect on plasmid pUB307 (IncP), which recorded a transfer frequency of 72.06*%* ± 33.42*%*. Interestingly, azithromycin (**3**) significantly enhanced the transfer of plasmid R7K (IncW), increasing its transfer frequency to 180.06*%* ± 81.23*%* (Figure 1). The inhibitory activity observed against plasmid pKM101 (IncN) is consistent with findings from a recent study by Jia et al. [35], which reported that azithromycin possesses broad‐spectrum horizontal transfer inhibition against c plasmids carrying clinically important antibiotic resistance genes including *mcr-1*, *bla*_NDM-5_, and *tet*(X4) [35]. Mechanistic investigations from the study demonstrated that azithromycin suppresses both intragenus and intergenus plasmid conjugation by downregulating bacterial secretion systems required for conjugative pilus formation. Furthermore, thymidine kinase, an enzyme critical for DNA synthesis, was identified as a potential target of azithromycin. The observed paradoxical effect of azithromycin (**3**) against plasmids pUB307 and R7K may be due to azithromycin‐induced bacterial stress response or physiological changes that favor conjugation.

Clarithromycin (**4**), at a concentration of 1 mg/L, actively inhibited the conjugal transfer of plasmid pKM101 (IncN) and moderately inhibited the transfer of plasmid R7K (IncW), with no significant plasmid curing activity observed for any of the test plasmids (Figure 2). The conjugation frequencies were reduced to 5.36*%* ± 4.64*%* for pKM101 and 31.25*%* ± 8.60*%* for R7K, respectively (Figure 1). Clarithromycin, like other macrolides, exerts its antibacterial activity by binding to the 50S ribosomal subunit and inhibiting bacterial protein synthesis. This disruption of protein synthesis may interfere with the cellular machinery for conjugation, thereby explaining its observed anticonjugant activity. A similar mechanism may underlie the broad‐spectrum horizontal transfer inhibition reported for azithromycin, another macrolide, by Jia et al. [35].

Doxycycline (**5**), at a concentration of 0.031 mg/L, exhibited pronounced plasmid curing activity, eliminating plasmids with the following efficiencies: 37.15*%* ± 13.41*%* for plasmid R7K (IncW), 17.38*%* ± 2.95*%* for pUB307 (IncP), and 6.57*%* ± 3.81*%* for pKM101 (IncN) (Figure 2). Interestingly, despite its plasmid‐curing effect, doxycycline was observed to enhance the conjugative transfer of R7K (IncW) with a transfer frequency of 126.05*%* ± 7.88*%* (Figure 1). This paradoxical increase might be attributed to doxycycline‐induced bacterial stress response or physiological changes that promote conjugation. Conversely, complete inhibition of conjugation was noted for plasmid pKM101 (IncN), which was statistically significant, and a moderate inhibitory effect of 26.07*%* ± 17.16*%* was observed for pUB307 (IncP) (Figure 1). These anticonjugant effects may be linked to the plasmid elimination (Figure 2) and doxycycline′s mechanism of action as a tetracycline antibiotic, which binds to the 30S ribosomal subunit, thereby inhibiting protein synthesis, an essential step in bacterial conjugation.

Ceftriaxone (**6**) at a concentration of 0.125 mg/L exhibited no marked plasmid curing effect (Figure 2) yet caused a significant reduction in the conjugative transfer of plasmids pKM101 (IncN), reducing transfer frequency to 6.05*%* ± 5.46*%*. Moreover, it completely inhibited the conjugation of plasmid R7K (IncW) (Figure 1), which was statistically significant. Ceftriaxone functions by inhibiting bacterial cell wall synthesis through binding to penicillin‐binding proteins. Although the cell wall is not directly involved in the conjugation process, as protein synthesis is, it provides structural support and anchorage for the conjugative pilus. This role may account for the observed anticonjugant activity of ceftriaxone in this study. Interestingly, in the presence of plasmid pUB307 (IncP), ceftriaxone enhanced conjugation, with a transfer frequency exceeding 120%. A plausible explanation for this effect is that subinhibitory concentrations of ceftriaxone may trigger bacterial envelope stress response systems, which in turn upregulate genes involved in membrane repair, remodeling, and activation of *tra* genes responsible for conjugative pili formation [32, 36].

At a concentration of 64 mg/L, glibenclamide (**7**) showed broad‐spectrum anticonjugant activity against all test plasmids. It moderately reduced the conjugation frequencies of plasmids pKM101 (IncN) and pUB (IncP) to 57.81*%* ± 22.44*%* and 25.05*%* ± 16.40*%*, respectively, and completely inhibited the conjugation of plasmid R7K (IncW), recording a frequency of 0, which was statistically significant (Figure 1). In addition, glibenclamide demonstrated plasmid‐curing activity against all test plasmids (Figure 2), which may account for the observed reduction in conjugation frequency. This effect could be linked to its known anti‐inflammatory property, which may modulate stress responses in the environment [37]. Beyond its primary role in stimulating insulin secretion from pancreatic *β*‐cells, glibenclamide has been reported to exert strong anti‐inflammatory effects by reducing the secretion of macrophage‐derived proinflammatory cytokines such as IL‐1*β* and IL‐18 [38]. These actions are associated with a reduction in oxidative stress and diminished reactive oxygen species (ROS) formation, factors that may influence plasmid stability and conjugative transfer.

Levofloxacin (**8**) at a concentration of 1 mg/L exhibited broad‐spectrum anticonjugant activity across all test plasmids. It significantly reduced the conjugative transfer frequencies of plasmids pKM101 (IncN) and R7K (IncW) to 5.35*%* ± 4.77*%* and 10.26*%* ± 5.84*%*, respectively. A moderately inhibitory effect was also observed for plasmid pUB307 (IncW), which recorded a conjugation frequency of 32.60*%* ± 18.45*%* (Figure 1). Furthermore, levofloxacin demonstrated plasmid‐curing activity against all test plasmids (Figure 2), which likely contributed to the observed reduction in conjugative transfer. Mechanistically, as a quinolone derivative, levofloxacin likely inhibits plasmid transfer by binding to bacterial DNA gyrase and topoisomerase IV, thereby disrupting DNA replication processes essential for plasmid maintenance and transfer. This aligns with findings by Michel‐Briand et al. [39], who reported that various quinolone derivatives including ciprofloxacin, cinoxacin, enoxacin, norfloxacin, flumequine, nalidixic acid, oxolinic acid, perfloxacin, pipemidic acid, rosoxacin, piromidic, beta‐hydroxypiromidic, beta‐hydroxypiromidic acid, and novobiocin exhibited plasmid‐curing activity at subinhibitory concentrations in *Enterobacteriaceae* species. Interestingly, these results contrast with those of Shun‐Mei et al. [32], who found that subinhibitory concentrations of ciprofloxacin and levofloxacin markedly increased the transfer frequency of the RP4 plasmid from *E. coli* to *Pseudomonas* species. This discrepancy may be attributed to variations in experimental conditions, bacterial host species, or plasmid types used in the studies.

In all, this study showed the specificity and complexity of plasmid‐mediated conjugation, where test samples may inhibit one plasmid but not the other or showed broad‐spectrum inhibition with or without plasmid elimination. The observations in this study align with previous findings that reported the specificity of HGT events depending on plasmid types, host factors, and environmental conditions [7, 40–42]. It further corroborates evidence that nonantibiotic pharmaceuticals, including cardiovascular drugs like amlodipine and antidiabetic drugs like glibenclamide, can facilitate HGT, thereby contributing to the spread of antimicrobial resistance genes via conjugation or transformation [17, 31]. Also, while prior studies have demonstrated that nonantibiotic pharmaceuticals can modulate bacterial physiology and HGT [14, 15, 17], the current study provides a more complete, plasmid‐aware functional assessment by using a combination of MIC determination, subinhibitory conjugation assays, and quantitative plasmid‐curing measurements employing plasmids belonging to three distinct incompatibility groups (IncN, IncP, and IncW).

Propranolol and amlodipine were observed to promote plasmid‐mediated conjugation of pKM101 (IncN) and pUB307 (IncP). This is consistent with previous studies that reported that certain cardiovascular, NSAIDS, and psychotropic drugs promoted conjugation. However, our study also observed pronounced plasmid‐specific inhibition and curing (e.g., amlodipine and glibenclamide strongly cured and completely inhibited conjugation of R7K, IncW). These drugs exhibited plasmid‐specific outcomes, where a single compound can both enhance the transfer of some plasmids and eliminate others—providing some clarity on certain reported contradictions in the literature. For instance, quinolones have been reported to both promote and/or inhibit plasmid transfer at subinhibitory concentrations depending on the host or plasmids type [16, 32].

By reporting both explicit curing percentages and transfer frequencies, our study distinguishes whether reduced transfer results from active inhibition of conjugation machinery or from plasmid loss, and it aligns our in vitro observations with recent population‐level evidence that commonly used nonantibiotic medications influence AMR development in *E. coli* [7].

This calls for increased awareness of the ecological impact of commonly used drugs beyond their primary therapeutic targets, as recent evidence has shown that widely prescribed nonantibiotic medications can directly influence antimicrobial resistance development in *E. coli* [7].

### 4.1. Conclusions

At subinhibitory concentrations, several antibiotics, including azithromycin, doxycycline, and ceftriaxone as well as nonantibiotic pharmaceuticals such as amlodipine and propranolol, promoted the horizontal transfer of plasmid‐borne antibiotic‐resistant genes in a plasmid‐specific manner. Clarithromycin demonstrated selective inhibitory effects on plasmid conjugation, whereas glibenclamide and levofloxacin exhibited broad‐spectrum anticonjugal activity across all test plasmids. Plasmid‐curing activity was also observed to be broad‐spectrum/nonselective for amlodipine, doxycycline, glibenclamide, and levofloxacin, whereas propranolol exhibited plasmid‐specificity curing activity, notably against plasmid R7K (IncW). These findings demonstrate that antibiotics and nonantibiotic drugs can exert dual, context‐dependent effects, simultaneously promoting plasmid transfer and eliminating specific plasmids. This plasmid‐specific interplay highlights the complex and variable influence of both antibiotics and nonantibiotic agents on HGT and emphasizes the need for further exploration of the ecological and clinical implications.

In conclusion, nonantibiotic pharmaceuticals are not benign in microbial ecology, suggesting that routine medications may inadvertently contribute to AMR through plasmid‐mediated conjugation.

### 4.2. Limitations

Limitations of this study include the use of reference strain *E. coli* ATCC 25922 for MIC determination rather than the donor and recipient strains used in the conjugation assays. While the use of standardized quality‐control strain ensures reproducibility and alignment with established guidelines, MIC values are known to be strain‐dependent and may be influenced by factors such as plasmid carriage, metabolic burden, and intrinsic resistance mechanisms. Future studies should therefore include MIC determination for all experimental strains to refine concentration selection and better account for strain‐specific response. Additionally, the use of laboratory‐adapted *E. coli* strains and model plasmids (pUB307 and R7K) may vary from clinical isolates and therefore may not precisely predict the impact of most antibiotic and nonantibiotic drugs on the transmission of AMR plasmids in a clinical setting. The use of only *E. coli* poses another limitation to the applicability of this data in a clinical setting.

## Author Contributions

**Marcus Daitey Larnyoh**, **Seth Kwabena Amponsah**, and **Awo Afi Kwapong** conceived the study. **Marcus Daitey Larnyoh**, **Seth Kwabena Amponsah**, **Abigail Offei**, **Emmanuel Kwaku Ofori**, **Ofosua Adi-Dako**, and **Awo Afi Kwapong** designed the methodology. **Marcus Daitey Larnyoh** and **Abigail Offei** performed the experiments under the supervision of **Seth Kwabena Amponsah**, **Emmanuel Kwaku Ofori**, **Ofosua Adi-Dako**, and **Awo Afi Kwapong**. **Marcus Daitey Larnyoh**, **Abigail Offei**, and **Awo Afi Kwapong** summarized data. **Marcus Daitey Larnyoh**, **Seth Kwabena Amponsah**, **Abigail Offei**, **Emmanuel Kwaku Ofori**, **Ofosua Adi-Dako**, and **Awo Afi Kwapong** were involved in the analysis of data and writing of the manuscript.

## Funding

No funding was received for this manuscript.

## Disclosure

All authors read and approved the final manuscript.

## Ethics Statement

The ethical and protocol review committee of the College of Health Sciences of the University of Ghana Medical School reviewed and approved the research proposal (Protocol ID: CHS‐Et/M.7‐P 4.3/2022‐2023). All experiments were carried out following good laboratory practices.

## Conflicts of Interest

The authors declare no conflicts of interest.

## Supporting information


**Supporting Information** Additional supporting information can be found online in the Supporting Information section. Table S1: This table reports the colony‐forming units per milliliter (CFU/mL) of donor and recipient cells used for the plasmid conjugation inhibition assay. The data shows the starting cell densities for evaluating the effect of the test samples. Table S2: This table presents the ratio of transconjugant cells to donor cells following conjugation in the presence of the selected antibiotics, nonantibiotics, and no‐drug control. The assay evaluates the effect of these agents on the conjugal transfer efficiency of IncN plasmid pKM101. Results are expressed as colony‐forming units per milliliter (CFU/mL), highlighting variations in plasmid transfer rates across conditions. Table S3: This table presents the ratio of transconjugant cells to donor cells following conjugation in the presence of the selected antibiotics, nonantibiotics, and no‐drug control. The assay evaluates the effect of these agents on the conjugal transfer efficiency of IncP plasmid pUB307. Results are expressed as colony‐forming units per milliliter (CFU/mL), highlighting variations in plasmid transfer rates across conditions. Table S4: This table presents the ratio of transconjugant cells to donor cells following conjugation in the presence of the selected antibiotics, nonantibiotics, and no‐drug control. The assay evaluates the effect of these agents on the conjugal transfer efficiency of IncW plasmid R7K. Results are expressed as colony‐forming units per milliliter (CFU/mL), highlighting variations in plasmid transfer rates across conditions. Table S5: This table reports the total colony‐forming unit per milliliter (CFU/mL) of bacterial cells and CFU/mL of plasmid‐bearing cells following the elimination assay conducted under different treatment conditions. These conditions include exposure to selected antibiotics, nonantibiotic agents, and a no‐drug control. The assay evaluates the impact of these agents on the maintenance or elimination of the IncN plasmid pKM101 within the bacterial population. Table S6: This table reports the total colony‐forming unit per milliliter (CFU/mL) of bacterial cells and CFU/mL of plasmid‐bearing cells following the elimination assay conducted under different treatment conditions. These conditions include exposure to selected antibiotics, nonantibiotic agents, and a no‐drug control. The assay evaluates the impact of these agents on the maintenance or elimination of the IncP plasmid pUB307 within the bacterial population. Table S7: This table reports the total colony‐forming unit per milliliter (CFU/mL) of bacterial cells and CFU/mL of plasmid‐bearing cells following the elimination assay conducted under different treatment conditions. These conditions include exposure to selected antibiotics, nonantibiotic agents, and a no‐drug control. The assay evaluates the impact of these agents on the maintenance or elimination of the IncW plasmid R7K within the bacterial population. Figure S1: Representative MTT assay plates showing colour change used to determine minimum inhibitory concentrations (MICs) of antibiotics and nonantibiotic drugs against *E. coli* ATCC 25922.

## Data Availability

Data from the current study are available upon request from corresponding authors.
