# Supplementary material for: The Effect of Antibiotic and Nonantibiotic Drugs on Plasmid‐Mediated Bacterial Conjugation
Source: Int J Microbiol. 2026 May 6;2026:3323758. doi: 10.1155/ijm/3323758 (PMC13149229; doi:10.1155/ijm/3323758)

**SUPPLEMENTARY DATA**

**Table S 1**: The colony-forming units per milliliters of donor and recipient cells used for the plasmid conjugation inhibition assay

| **Plasmid (Sample ID)** | | **Dilution Factor** | **Colony Forming Unit (CFU)** | | **Average CFU** | **CFU/mL** |
| --- | --- | --- | --- | --- | --- | --- |
|  |  |  | **Plate 1** | **Plate 2** |  |  |
| Donor | pKM101 | 10^-6^ | 195 | 199 | 197 | 9.85 x 10^9^ |
|  |  | 10^-7^ | 44 | 48 | 46 | 2.30 x 10^10^ |
|  | pUB307 | 10^-5^ | 284 | 288 | 286 | 1.43 x 10^9^ |
|  |  | 10^-6^ | 42 | 46 | 44 | 2.20 x 10^9^ |
|  |  | 10^-7^ | 35 | 39 | 37 | 1.85 x 10^9^ |
|  | R7K | 10^-6^ | 255 | 257 | 256 | 1.28 x 10^10^ |
|  |  | 10^-7^ | 36 | 40 | 38 | 1.90 x 10^10^ |
| Recipient | ER1793 | 10^-4^ | 272 | 276 | 274 | 1.37 x 10^8^ |
|  |  | 10^-5^ | 52 | 48 | 50 | 2.50 x 10^8^ |
|  |  | 10^-6^ | 33 | 37 | 35 | 1.75 x 10^9^ |
|  |  | 10^-7^ | 12 | 8 | 10 | 5.00 x 10^9^ |
|  | JM109 | 10^-4^ | 40 | 56 | 48 | 2.40 x 10^7^ |
|  |  | 10^-5^ | 12 | 34 | 23 | 1.15 x 10^8^ |
|  |  | 10^-6^ | 20 | 14 | 17 | 8.50 x 10^8^ |
|  |  | 10^-7^ | 6 | 12 | 9 | - 1. x 10^9^ |

*Volume plated is 20 μL

CFU/mL calculation $=(\frac{average colony forming unit}{volume plated}) \times dilution factor$

**Table S 2**: Effect of selected antibiotics and non-antibiotics on conjugal transfer of IncN plasmid pKM101

| Test sample (Code) | Conjugation Frequency (CFU/mL) | | |
| --- | --- | --- | --- |
|  | Exp 1 | Exp 2 | Exp 3 |
| Control (Clt) | 1.83 x 10^-2^ | 4.13 x 10^-2^ | 5.13 x 10^-2^ |
| Amlodipine (1) | 5.71 x 10^-2^ | 8.33 x 10^-2^ | 1.25 x 10^-1^ |
| Propranolol (2) | 2.38 x 10^-2^ | 4.00 x 10^-2^ | 6.00 x 10^-2^ |
| Azithromycin (3) | 1.55 x 10^-3^ | 2.56 x 10^-3^ | 0 |
| Clarithromycin (4) | 1.51 x 10^-3^ | 3.25 x 10^-3^ | 0 |
| Doxycycline (5) | 0 | 0 | 0 |
| Ceftriaxone (6) | 1.95 x 10^-3^ | 3.12 x 10^-3^ | 0 |
| Glibenclamide (7) | 1.50 x 10^-2^ | 2.23 x 10^-2^ | 1.92 x 10^-2^ |
| Levofloxacin (8) | 1.68 x 10^-3^ | 2.84 x 10^-3^ | 0 |

Clt – control without test compound/ no drug

**Table S 3:** Effect of selected antibiotics and non-antibiotics on conjugal transfer of IncP plasmid pUB307

| Test sample (Code) | Conjugation Frequency (CFU/mL) | | |
| --- | --- | --- | --- |
|  | Exp 1 | Exp 2 | Exp 3 |
| Control (Clt) | 2.50 x 10^-2^ | 1.95 x 10^-2^ | 7.69 x 10^-2^ |
| Amlodipine (1) | 1.13 x 10^-1^ | 1.31 x 10^-1^ | 1.10 x 10^-1^ |
| Propranolol (2) | 3.30 x 10^-2^ | 3.13 x 10^-2^ | 3.49 x 10^-2^ |
| Azithromycin (3) | 2.11 x 10^-2^ | 1.90 x 10^-2^ | 2.63 x 10^-2^ |
| Clarithromycin (4) | 3.33 x 10^-2^ | 2.49 x 10^-2^ | 2.81 x 10^-2^ |
| Doxycycline (5) | 9.22 x 10^-3^ | 6.85 x 10^-3^ | 4.83 x 10^-3^ |
| Ceftriaxone (6) | 9.48 x 10^-2^ | 8.52 x 10^-2^ | 1.32 x 10^-1^ |
| Glibenclamide (7) | 9.52 x 10^-3^ | 5.94 x 10^-3^ | 5.10 x 10^-3^ |
| Levofloxacin (8) | 1.17 x 10^-2^ | 7.69 x 10^-3^ | 9.01 x 10^-3^ |

Clt – control without test compound/ no drug

**Table S 4**: Effect of selected antibiotics and non-antibiotics on conjugal transfer of IncW plasmid R7K

| Test sample (Code) | Conjugation Frequency (CFU/mL) | | |
| --- | --- | --- | --- |
|  | Exp 1 | Exp 2 | Exp 3 |
| Control (Clt) | 1.38 x 10^0^ | 5.67 x 10^-2^ | 2.09 x 10^-2^ |
| Amlodipine (1) | 0 | 0 | 0 |
| Propranolol (2) | 4.57 x 10^-1^ | 3.23 x 10^-1^ | 6.25 x 10^-2^ |
| Azithromycin (3) | 1.21 x 10^0^ | 1.22 x 10^0^ | 5.00 x 10^-1^ |
| Clarithromycin (4) | 5.30 x 10^-1^ | 1.23 x 10^-1^ | 7.02 x 10^-2^ |
| Doxycycline (5) | 1.76 x 10^0^ | 6.67 x 10^-1^ | 2.78 x 10^-1^ |
| Ceftriaxone (6) | 0. | 0 | 0 |
| Glibenclamide (7) | 0 | 0 | 0 |
| Levofloxacin (8) | 5.52 x 10^-2^ | 6.36 x 10^-2^ | 3.26 x 10^-2^ |

Clt – control without test compound/ no drug

**Table S 5**: Plasmid elimination effect of the test samples on IncN plasmid pKM101

| Test sample (Code) | Total colony forming unit/mL | | | Colony forming unit of plasmid-bearing cells/mL | | |
| --- | --- | --- | --- | --- | --- | --- |
|  | Exp 1 | Exp 2 | Exp 3 | Exp 1 | Exp 2 | Exp 3 |
| Control (Clt) | 6.20 x 10^7^ | 5.70 x 10^7^ | 6.70 x 10^7^ | 6.05 x 10^7^ | 5.50 x 10^7^ | 6.40 x 10^7^ |
| Amlodipine (1) | 4.20 x 10^7^ | 3.65 x 10^7^ | 3.40 x 10^7^ | 3.00 x 10^7^ | 2.90 x 10^7^ | 2.40 x 10^7^ |
| Propranolol (2) | 5.20 x 10^7^ | 5.50 x 10^7^ | 5.00 x 10^7^ | 5.00 x 10^7^ | 5.20 x 10^7^ | 4.90 x 10^7^ |
| Azithromycin (3) | 1.99 x 10^8^ | 1.80 x 10^8^ | 1.91 x 10^8^ | 1.95 x 10^8^ | 1.78 x 10^8^ | 1.91 x 10^8^ |
| Clarithromycin (4) | 1.57 x 10^8^ | 1.41 x 10^8^ | 1.42 x 10^8^ | 1.54 x 10^8^ | 1.39 x 10^8^ | 1.40 x 10^8^ |
| Doxycycline (5) | 1.19 x 10^8^ | 1.14 x 10^8^ | 1.10 x 10^8^ | 1.14 x 10^8^ | 1.02 x 10^8^ | 1.05 x 10^8^ |
| Ceftriaxone (6) | 1.64 x 10^8^ | 1.56 x 10^8^ | 1.62 x 10^8^ | 1.61 x 10^8^ | 1.54 x 10^8^ | 1.60 x 10^8^ |
| Glibenclamide (7) | 1.19 x 10^8^ | 1.17 x 10^8^ | 1.15 x 108 | 1.12 x 10^8^ | 1.09 x 10^8^ | 1.02 x 10^8^ |
| Levofloxacin (8) | 1.91 x 10^8^ | 1.93 x 10^8^ | 1.94 x 10^8^ | 1.76 x 10^8^ | 1.75 x 10^8^ | 1.70 x 10^8^ |
| Promethazine (Pmz) | 1.25 x 10^8^ | 1.22 x 10^8^ | 1.25 x 10^8^ | 1.04 x 10^8^ | 9.90 x 10^7^ | 1.00 x 10^8^ |

The negative control Clt (without test sample/ no drug), and the positive control promethazine (Pmz).

**Table S 6**: Plasmid elimination effect of the test samples on IncP plasmid pUB307

| Test sample (Code) | Total colony forming unit | | | Colony forming unit of plasmid-bearing cells | | |
| --- | --- | --- | --- | --- | --- | --- |
|  | Exp 1 | Exp 2 | Exp 3 | Exp 1 | Exp 2 | Exp 3 |
| Control (Clt) | 8.10 x 10^7^ | 7.50 x 10^7^ | 7.25 x10^7^ | 7.60 x 10^7^ | 7.50 x 10^7^ | 7.10 x 10^7^ |
| Amlodipine (1) | 9.00 x 10^7^ | 9.20 x 10^7^ | 9.40 x 10^7^ | 7.75 x 10^7^ | 6.75 x 107 | 6.95 x 10^7^ |
| Propranolol (2) | 1.73 x 10^8^ | 1.62 x 10^8^ | 1.65 x 10^8^ | 1.72 x 10^8^ | 1.62 x 10^8^ | 1.60 x 10^8^ |
| Azithromycin (3) | 1.60 x 10^8^ | 1.49 x 10^8^ | 1.49 x 10^8^ | 1.52 x 10^8^ | 1.49 x 10^8^ | 1.44 x 10^8^ |
| Clarithromycin (4) | 1.85 x 10^8^ | 1.79 x 10^8^ | 1.85 x 10^8^ | 1.85 x 10^8^ | 1.78 x 10^8^ | 1.84 x 10^8^ |
| Doxycycline (5) | 1.21 x 10^8^ | 1.24 x 10^8^ | 1.21 x 10^8^ | 1.04 x 10^8^ | 9.90 x 10^7^ | 9.90 x 10^7^ |
| Ceftriaxone (6) | 5.50 x 10^7^ | 5.00 x 10^7^ | 5.10 x 10^7^ | 5.30 x 10^7^ | 4.90 x 10^7^ | 5.10 x 10^7^ |
| Glibenclamide (7) | 1.15 x 10^8^ | 1.21 x 10^8^ | 1.30 x 10^8^ | 9.80 x 10^7^ | 9.45x 10^7^ | 9.90 x 10^7^ |
| Levofloxacin (8) | 8.00 x 10^6^ | 1.40 x 10^7^ | 1.00 x 10^7^ | 5.50 x 10^6^ | 8.00 x 10^6^ | 9.00 x 10^6^ |
| Promethazine (Pmz) | 7.40 x 10^7^ | 7.10 x 10^7^ | 7.40 x 10^7^ | 5.60 x 10^7^ | 5.40 x 10^7^ | 5.10 x 10^7^ |

The negative control Clt (without test sample/ no drug), and the positive control promethazine (Pmz).

**Table S 7**: Plasmid elimination effect of the test samples on IncW plasmid R7K

| Test sample (Code) | Total colony forming unit | | | Colony forming unit of plasmid-bearing cells | | |
| --- | --- | --- | --- | --- | --- | --- |
|  | Exp 1 | Exp 2 | Exp 3 | Exp 1 | Exp 2 | Exp 3 |
| Control (Clt) | 1.25 x 10^10^ | 1.10 x 10^11^ | 1.06 x 10^11^ | 1.23 x 10^10^ | 1.08 x 10^11^ | 1.05 x 10^11^ |
| Amlodipine (1) | 3.75 x 10^9^ | 2.65 x 10^10^ | 2.90 x 10^10^ | 3.45 x 10^9^ | 1.50 x 10^10^ | 1.40 x 10^10^ |
| Propranolol (2) | 1.55 x10^9^ | 9.00 x 10^9^ | 9.00 x 10^9^ | 1.55 x 10^9^ | 8.00 x 10^9^ | 6.00 x 10^9^ |
| Azithromycin (3) | 3.55 x 10^9^ | 1.80 x 10^10^ | 1.50 x 10^10^ | 3.45 x 10^9^ | 1.80 x 10^10^ | 1.50 x 10^10^ |
| Clarithromycin (4) | 7.30 x 10^9^ | 5.90 x 10^10^ | 5.60 x 10^10^ | 7.30 x 10^9^ | 5.70 x 10^10^ | 5.40 x 10^10^ |
| Doxycycline (5) | 1.70 x 10^9^ | 1.90 x 10^10^ | 1.70 x 10^10^ | 1.20 x 10^9^ | 9.00 x 10^9^ | 1.20 x 10^10^ |
| Ceftriaxone (6) | 6.00 x 10^8^ | 2.00 x 10^9^ | 2.00 x 10^9^ | 5.00 x 10^8^ | 2.00 x 10^9^ | 2.00 x 10^9^ |
| Glibenclamide (7) | 5.05 x 10^9^ | 4.05 x 10^10^ | 4.15 x 10^10^ | 4.55 x 10^9^ | 2.85 x 10^10^ | 2.50 x 10^10^ |
| Levofloxacin (8) | 1.28 x 10^10^ | 1.28 x 10^11^ | 1.11 x 10^11^ | 1.18 x10^10^ | 1.08 x 10^11^ | 1.01 x 10^11^ |
| Promethazine (Pmz) | 9.30 x 10^9^ | 1.40 x10^10^ | 1.50 x 10^10^ | 7.25 x10^9^ | 7.50 x 10^9^ | 9.00 x 10^9^ |

The negative control Clt (without test sample/ no drug), and the positive control promethazine (Pmz).

Representative MTT assay plates showing colour change used to determine minimum inhibitory concentrations (MICs) of antibiotics and non-antibiotic drugs against *E. coli* ATCC 25922

Azithromycinn

Clarithromycin

Doxycycline

Control


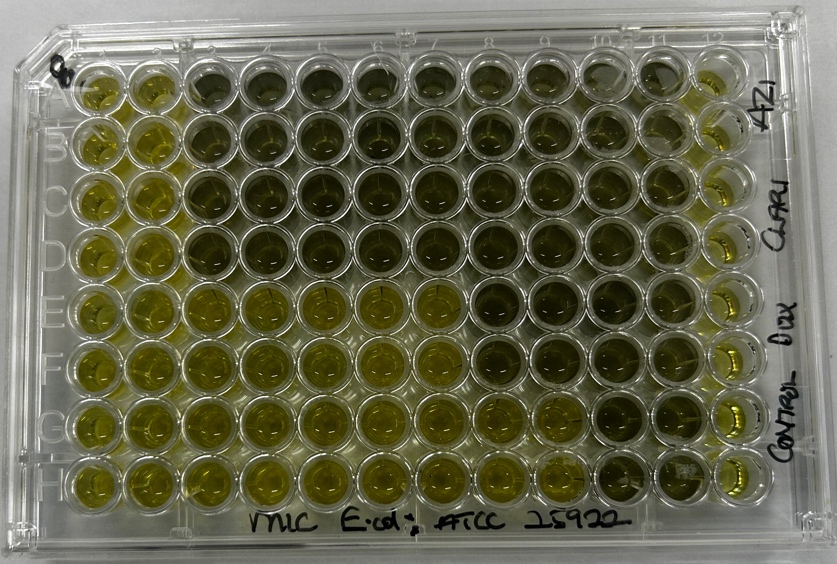

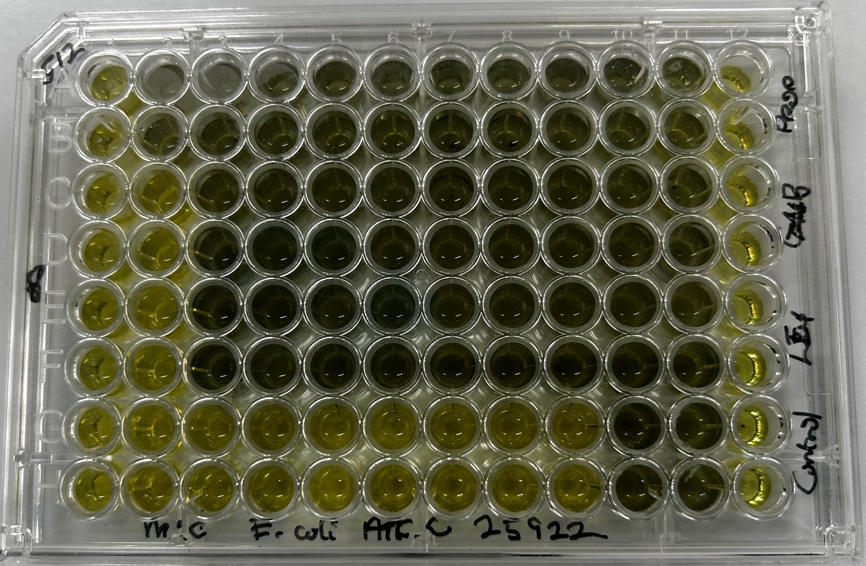


Propranolol

Glibenclamide

Levofloxacin

Control

Control

Cefixime

Cefuroxime

Ceftriaxone


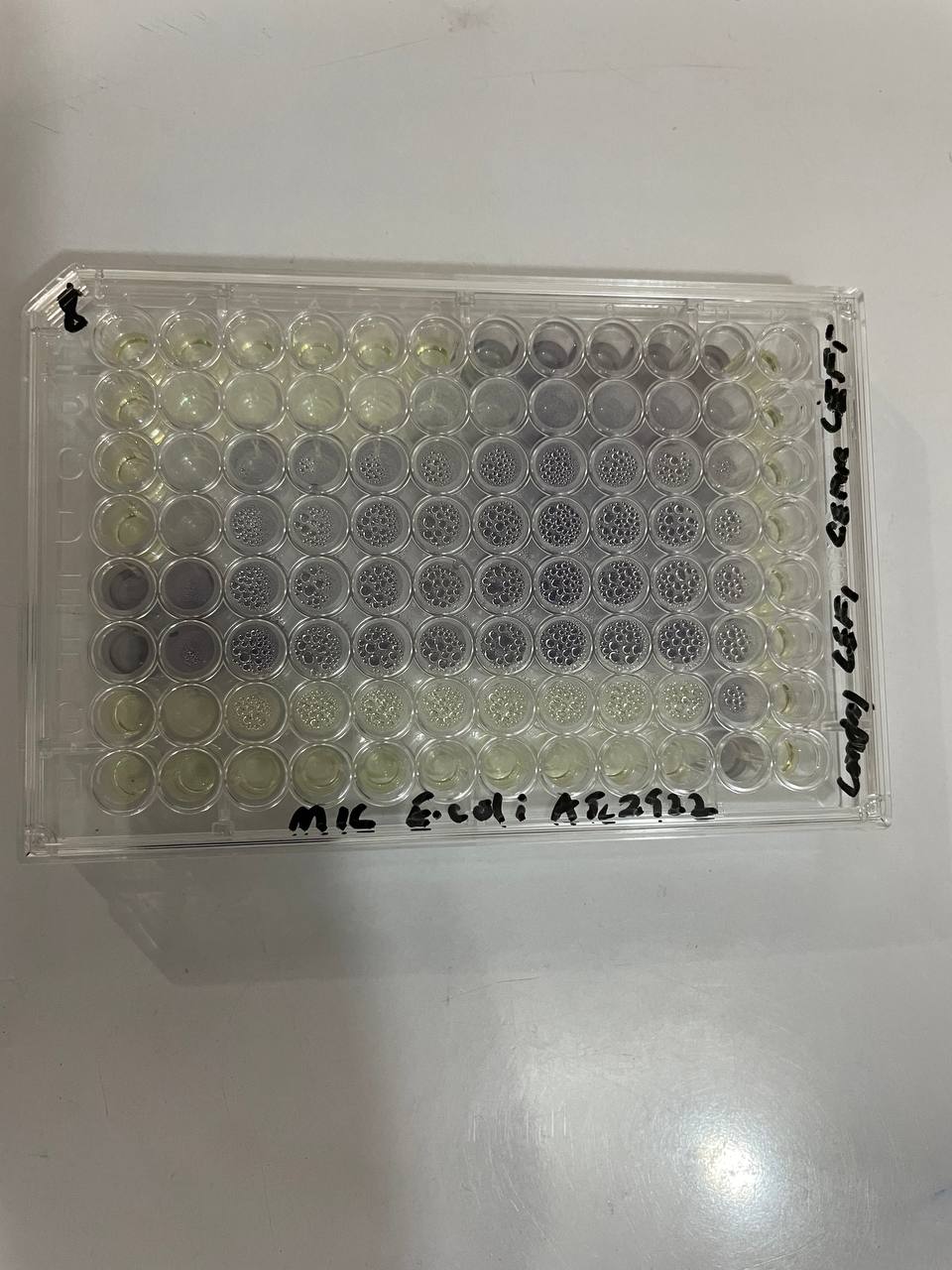


Amlodipine

Lisinopril

Methyldopa

Control


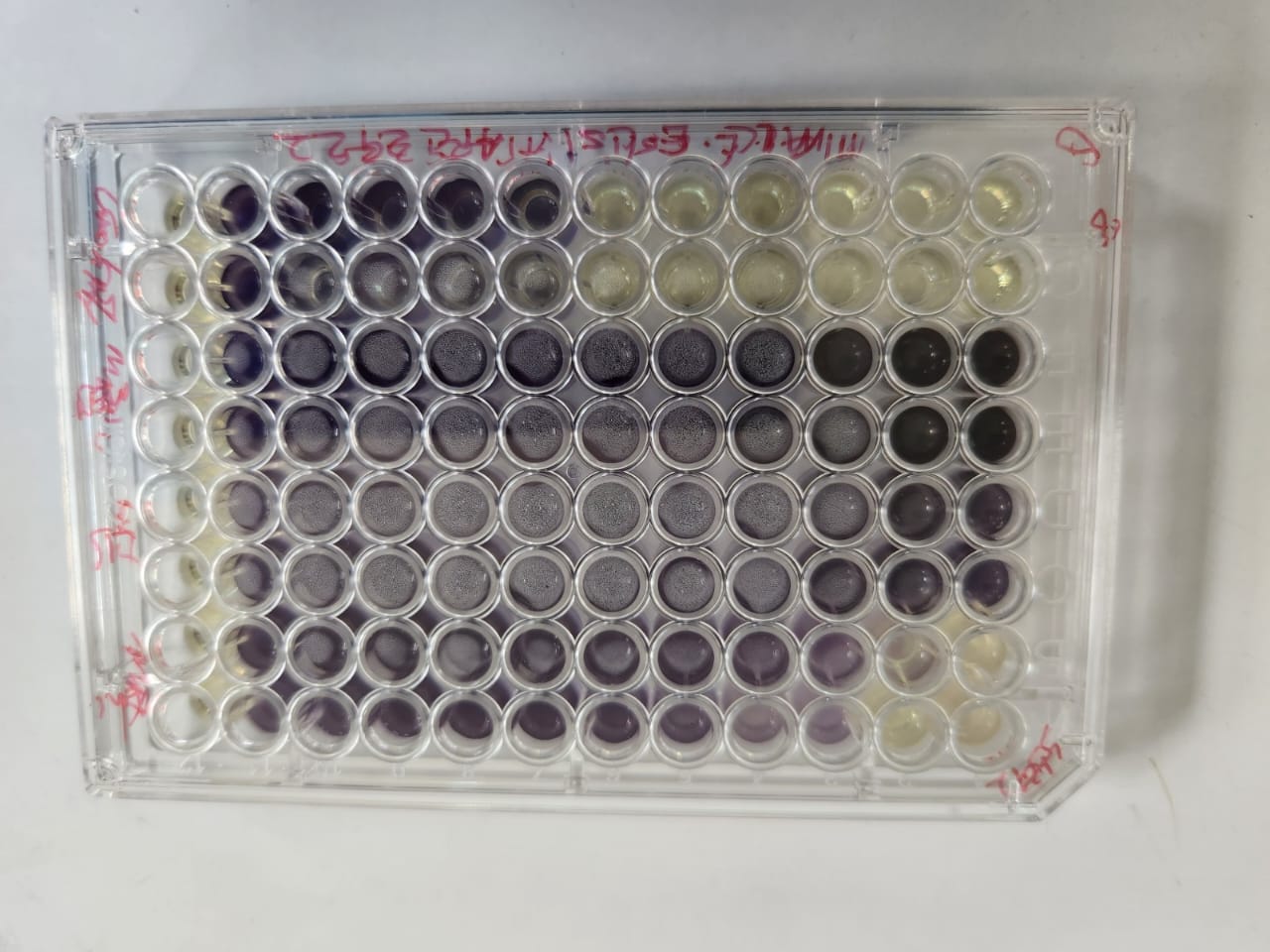


**Figure S1**: Supplementary images of 96–well microtiter plates showing Minimum Inhibitory Concentrations (MICs) of antibiotics and non-antibiotics against E. coli ATCC 25922. Wells with purple coloration indicate bacterial metabolic activity (growth), while yellow coloration indicates inhibition of bacterial growth. The starting concentrations (512 or 8 mg/L) and names of antibiotics and non-antibiotics are indicated on the plates. The control drug used was ciprofloxacin, with a starting concentration of 8 mg/L on each plate. A two-fold (serial) dilution scheme was used on each plate.


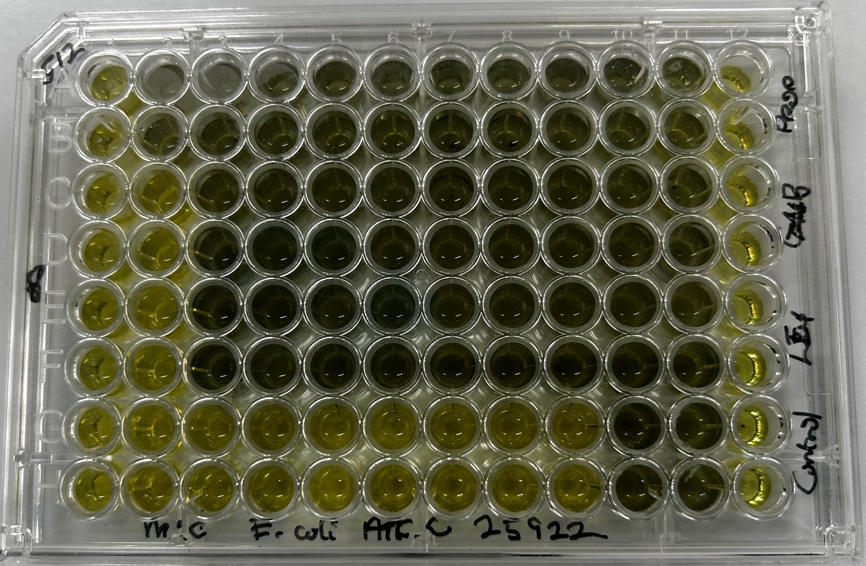

Supplement: Supplementary file 1 — Supporting Information Additional supporting information can be found online in the Supporting Information section. Table S1: This table reports the colony‐forming units per milliliter (CFU/mL) of donor and recipient cells used for the plasmid conjugation inhibition assay. The data shows the starting cell densities for evaluating the effect of the test samples. Table S2: This table presents the ratio of transconjugant cells to donor cells following conjugation in the presence of the selected antibiotics, nonantibiotics, and no‐drug control. The assay evaluates the effect of these agents on the conjugal transfer efficiency of IncN plasmid pKM101. Results are expressed as colony‐forming units per milliliter (CFU/mL), highlighting variations in plasmid transfer rates across conditions. Table S3: This table presents the ratio of transconjugant cells to donor cells following conjugation in the presence of the selected antibiotics, nonantibiotics, and no‐drug control. The assay evaluates the effect of these agents on the conjugal transfer efficiency of IncP plasmid pUB307. Results are expressed as colony‐forming units per milliliter (CFU/mL), highlighting variations in plasmid transfer rates across conditions. Table S4: This table presents the ratio of transconjugant cells to donor cells following conjugation in the presence of the selected antibiotics, nonantibiotics, and no‐drug control. The assay evaluates the effect of these agents on the conjugal transfer efficiency of IncW plasmid R7K. Results are expressed as colony‐forming units per milliliter (CFU/mL), highlighting variations in plasmid transfer rates across conditions. Table S5: This table reports the total colony‐forming unit per milliliter (CFU/mL) of bacterial cells and CFU/mL of plasmid‐bearing cells following the elimination assay conducted under different treatment conditions. These conditions include exposure to selected antibiotics, nonantibiotic agents, and a no‐drug control. The assay evaluates the imp [file IJM-2026-3323758-s001.docx]
